# Cuidados com o acesso vascular para hemodiálise: revisão integrativa

**DOI:** 10.15649/cuidarte.2090

**Published:** 2021-09-27

**Authors:** Gabriela Araújo-Rocha, Ana Karoline Lima-de-Oliveira, Francisco Gerlai Lima-Oliveira, Vitória Eduarda Silva-Rodrigues, Antônio Gabriel de-Sousa-Moura, Evelton Barros-Sousa, Ana Larissa Gomes-Machado

**Affiliations:** 1 Universidade Federal do Piauí. Picos, PI, Brasil. E-mail: gabrielaaraujorocha@hotmail.com. Autor correspondente. Universidade Federal do Piauí Universidade Federal do Piauí PI Brazil gabrielaaraujorocha@hotmail.com.; 2 Universidade Federal do Piauí. Picos, PI, Brasil. E-mail: kcollarhes.kc@gmail.com. Universidade Federal do Piauí Universidade Federal do Piauí Picos Brazil kcollarhes.kc@gmail.com; 3 Universidade Federal do Piauí. Picos, PI, Brasil. E-mail: gerlailima@gmail.com. Universidade Federal do Piauí Universidade Federal do Piauí Picos PI Brazil gerlailima@gmail.com; 4 Universidade Federal do Piauí. Picos, PI, Brasil. E-mail: vittoriaeduarda@hotmail.com Universidade Federal do Piauí Universidade Federal do Piauí Picos PI Brazil vittoriaeduarda@hotmail.com; 5 Universidade Federal do Piauí. Picos, PI, Brasil. E-mail: gabrielmourasm@hotmail.com. Universidade Federal do Piauí Universidade Federal do Piauí Picos PI Brazil gabrielmourasm@hotmail.com; 6 Universidade Federal do Piauí. Picos, PI, Brasil. E-mail: eveltonb@gmail.com. Universidade Federal do Piauí Universidade Federal do Piauí Picos PI Brazil eveltonb@gmail.com; 7 Universidade Federal do Piauí. Picos, PI, Brasil. E-mail: analarissa2001@yahoo.com.br. Universidade Federal do Piauí Universidade Federal do Piauí Picos PI Brazil analarissa2001@yahoo.com.br

**Keywords:** Adulto, Diálise Renal, Fístula Arteriovenosa, Cateteres Venosos Centrais, Cuidados de Enfermagem., Adult, Renal Dialysis, Arteriovenous Fistula, Central Venous Catheters, Nursing Care., Adulto, Diálisis Renal, Fístula Arteriovenosa, Catéteres Venosos Centrales, Cuidado de Enfermeria.

## Abstract

**Introdução:**

As complicações relacionadas ao acesso vascular para hemodiálise podem resultar em intervenções complexas, contudo,cuidados adoptados pelos profissionais de saúde e pacientes adultos podem evitá-las. Objetivou-se analisar a produção científica acerca dos cuidados com acessos vasculares utilizados na hemodiálise para elaboração do conteúdo de uma cartilha educativa voltada ao autocuidado do paciente.

**Método:**

Revisão integrativa de literatura realizada nas bases de dados: LILACS, PUBMED, BDENF, SciELO e CINAHL. A busca dos estudos deu-se entre setembro e novembro de 2019, nos idiomas português, inglês ou espanhol, utilizando os termos dos DeCS e MeSH. A seleção dos artigos foi realizada por três pesquisadores e foram extraídas informações: país, ano de publicação, delineamento do estudo, número de pacientes, intervenções, desfechos e nível de evidência cinetífica. Selecionaram-se 10 artigos processados no *software* IRAMUTEQ® e analisados descritivamente pelo modelo de Reinert.

**Resultados:**

Organizaram-se sete classes: Cuidados com o cateter após a hemodiálise; Cuidados com a FAV antes da hemodiálise; Cuidados com a FAV após a hemodiálise; Cuidados para evitar a interrupção do funcionamento da FAV; Autocuidado dos pacientes com a FAV; Cuidados realizados pela equipe de enfermagem; Conhecimento do paciente acerca dos cuidados com a pele e punção da FAV.

**Conclusão:**

Identificou-se que os ciudados com os acessos vasculares mais frequentes se relacionam com a fístula arteriovenosa, demonstrando sua grande representatividade no tratamento do paciente com doença renal crônica. A síntese do conhecimento proporcionada nesta revisão foi utilizada para a elaboração de uma cartilha educativa já validada por especialistas e por pacientes que realizam hemodiálise.

## Abstract (en)

**Introduction:**

Complications related to vascular access for hemodialysis can result in complex interventions, however, care taken by health professionals and adult patients can avoid them. The objective was to analyze the scientific production about care with vascular access used in hemodialysis for the development of the content of an educational booklet aimed at patient self-care.

**Method:**

Integrative literature review carried out in the following databases: LILACS, PUBMED, BDENF, SciELO and CINAHL. The search for studies took place between September and November 2019, in Portuguese, English or Spanish, using the terms of DeCS and MeSH. The selection of articles was performed by three researchers and information was extracted: country, year of publication, study design, number of patients, interventions, outcomes and level of scientific evidence. Ten articles processed in the IRAMUTEQ® software and descriptively analyzed using the Reinert model were selected.

**Results:**

Seven classes were organized: Catheter care after hemodialysis; Care of the AVF before hemodialysis; Care with AVF after hemodialysis; Care to avoid interruption of AVF operation; Self-care of patients with AVF; Care performed by the nursing team; Patient knowledge about skin care and AVF puncture.

**Conclusion:**

It was identified that the care with the most frequent vascular accesses is related to the arteriovenous fistula, demonstrating its great representation in the treatment of patients with chronic kidney disease. The synthesis of knowledge provided in this review was used to prepare an educational booklet that has already been validated by specialists and by patients undergoing hemodialysis.

## Resumen

**Introducción:**

Las complicaciones relacionadas con el acceso vascular a la hemodiálisis pueden resultar en intervenciones complejas, sin embargo, los cuidados prestados por los profesionales de la salud y los pacientes adultos pueden evitarlas. El objetivo es analizar la producción científica sobre los cuidados con accesos vasculares utilizados en la hemodiálisis para la elaboración del contenido de una cartilla educativa para el autocuidado del paciente.

**Método:**

Revisión integrativa de la literatura realizada en las bases de datos: LILACS, PUBMED, BDENF, SciELO y CINAHL. La búsqueda de los estudios se realizó entre septiembre y noviembre de 2019, en los idiomas portugués, inglés o español, utilizando los términos del DeCS y MeSH. La selección de los artículos fue realizada por tres investigadores y se extrajo la información: país, año de publicación, diseño del estudio, número de pacientes, intervenciones, resultados y nivel de evidencia científica. Se seleccionaron diez artículos procesados en el software IRAMUTEQ® y analizados descriptivamente mediante el modelo de Reinert.

**Resultados:**

Se organizaron siete clases: Cuidados del catéter después de la hemodiálisis; Cuidados de la FAV antes de la hemodiálisis; Cuidados de la FAV después de la hemodiálisis; Cuidados para evitar la interrupción del funcionamiento de la FAV; Autocuidado del paciente con la FAV; Cuidados realizados por el equipo de enfermería; Conocimiento del paciente sobre el cuidado de la piel y la punción de la FAV.

**Conclusión:**

Se identificó que los cuidados con los accesos vasculares más frecuentes se relacionan con la fístula arteriovenosa, y su gran representatividad en el tratamiento del paciente con enfermedad renal crónica. La síntesis del conocimiento proporcionada en esta revisión se utilizó para la elaboración de una cartilla educativa ya validada por especialistas y por pacientes que realizan hemodiálisis.

## Introdução

As Doenças Crônicas Não Transmissíveis (DCNT) acarretam impactos negativos na saúde e qua- lidade de vida dos indivíduos, famílias e comunidades, bem como sobrecarregam os serviços de saúde, com onerosos gastos aos sistemas prestadores de serviço. Além disso, são as principais causas de incapacidade e mortalidade no mundo, em que 38 milhões de indivíduos morrem prematuramente(1),(2). A hipertensão e o diabetes são fatores associados à Doença Renal Crônica (DRC), sendo que esta apresenta prevalência na população adulta(3).

A DRC é um exemplo de DCNT que necessita de cuidados prolongados, pois está associada às alterações estruturais e progressivas da função renal, com redução da filtração glomerular. Essa condição de saúde requer acompanhamento por serviços especializados, tanto em seus estágios iniciais quanto finais, que requerem terapias renais substitutivas, como a hemodiálise (HD)(3).

O número total de pacientes em diálise crônica no Brasil, em 2019, foi estimado em 139.691, e destes, 93,2% estavam em hemodiálise(4). Para o sucesso da terapia renal substitutiva e o consequente bem- estar das pessoas com DRC, são fundamentais a confecção e a manutenção de um acesso vascular de qualidade, que forneça fluxo sanguíneo adequado, tenha boa durabilidade e apresente baixo risco de complicações(5).

Segundo o Inquérito Brasileiro de Diálise (2019),(4) o percentual calculado de pacientes em hemodiálise com acesso vascular do tipo fístula arteriovenosa (FAV) no Brasil é de 72,2%, seguido por cateter venoso central temporário, com 9,4%, e o permanente, que representa 15,4%. O uso de enxerto vascular (prótese) é de apenas 3%.

A literatura aponta a ocorrência de complicações relacionadas ao acesso vascular para hemodiálise, sendo as mais prevalentes a redução do fluxo sanguíneo, hemorragias e presença de infecção ou infecção instalada(6). Esses eventos adversos podem se agravar e resultar em intervenções mais complexas, como hospitalizações, bem como comprometer o funcionamento do acesso, necessitando de substituição. Essa problemática, no entanto, pode ser evitada e minimizada se os cuidados com o acesso venoso forem corretamente realizados pelos profissionais de saúde e pelo próprio paciente(7).

As práticas educativas envolvendo os pacientes adultos que realizam hemodiálise demonstram importância notável, principalmente acerca do autocuidado com os acessos vasculares, pois os clientes podem realizar ações, por vezes simples, porém de grande importância para a manutenção do acesso vascular funcionante a partir do conhecimento adquirido nas intervenções educativas. Ressalta-se ainda que os profissionais de saúde envolvidos na assistência devem transmitir informações através de uma comunicação clara e efetiva(8).

Nesse contexto, a equipe de enfermagem é fundamental na orientação dos pacientes adultos com DRC com relação às diversas mudanças nas atividades diárias, manutenção da autonomia e apoio psicológico. A promoção da saúde com o incentivo ao autocuidado leva a enfermagem a realizar um cuidado interativo, construído de forma conjunta e empoderado. A identificação e controle de possíveis complicações podem ser feitos também pelo paciente no domicílio, conferindo-lhe autonomia. Diante disso, o autocuidado assume particular importância, tendo em vista que um cliente bem orientado e preparado conseguirá desenvolver os cuidados necessários(9).

Mediante o exposto, o presente estudo teve como objetivo analisar a produção científica acerca dos cuidados com acessos vasculares utilizados na terapia hemodialítica para elaboração do conteúdo de uma cartilha educativa voltada ao autocuidado do paciente.

O estudo justifica-se pela necessidade de a pessoa em tratamento hemodialítico compreender o tratamento e os cuidados necessários para manter o seu acesso vascular funcionante, reduzindo intercorrências e prolongando os bons resultados do tratamento. Sua relevância consiste no esclarecimento das dúvidas relacionadas aos cuidados com os acessos vasculares, bem como ao corroborar a importância do papel do enfermeiro no âmbito educacional de orientação dos pacientes sobre os cuidados que devem ser realizados no serviço de saúde e no ambiente domiciliar.

## Método

### Tipo de estudo

Trata-se de uma revisão integrativa de literatura, desenvolvida em seis etapas: 1) Identificação do tema e seleção da hipótese; 2) Estabelecimento de critérios de inclusão e exclusão; 3) Categorização dos estudos selecionados; 4) Avaliação dos estudos selecionados; 5) Interpretação dos resultados e 6) Síntese do conhecimento(10). Esse tipo de revisão de literatura permite a síntese de múltiplos estudos publicados para a construção de uma análise ampla de literatura, de forma ordenada, sistemática e abrangente(11).

Elaborou-se a seguinte questão norteadora do estudo: “Quais são as evidências disponíveis na literatura sobre os cuidados com os acessos vasculares para hemodiálise realizada com pacientes adultos?”. Para orientar a elaboração da pergunta norteadora e a busca na literatura foi utilizada a estratégia PICO(12), conforme o Quadro 1.

**Quadro 1:** Elementos da estratégia PICO. Brasil, 2019

Acrônimo	Definição	Descrição
P	População de interesse	Pacientes adultos que realizam hemodiálise
I	Intervenção	Cuidados necessários com o acesso vascular
C	Comparação	Cuidados adequados
O	Resultados/desfechos	Acesso vascular para hemodiálise adequado e funcionante

Fonte: elaboração própria.

Os critérios de inclusão utilizados para a seleção dos artigos foram: artigo de pesquisa com texto completo disponível na íntegra, publicado nos idiomas português, inglês ou espanhol, divulgado entre 2013 e 2019, e que contemplassem os cuidados relacionados aos acessos vasculares utilizados para a hemodiálise. Como critério de exclusão adotaram-se abordagens que não respondiam à questão norteadora do estudo, bem como arquivos duplicados.

As bases de dados selecionadas para a pesquisa foram: Centro Latino-Americano e do Caribe de Informação em Ciências da Saúde (LILACS), PUBMED, Base de Dados em Enfermagem (BDENF), *Scientific Eletronic Library Online* (SciELO), e *Cumulative Index to Nursing and Allied Health Literature* (CINAHL).

**Figura 1:**
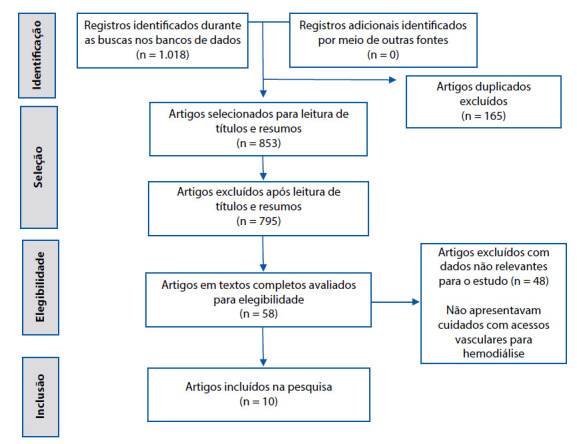
Fluxograma de seleção dos estudos primários, de acordo com a recomendação PRISMA. Brasil, 2019.

A operacionalização da pesquisa teve início com a consulta aos Descritores em Ciências da Saúde (DeCS) e *Medical Subject Headings* (MeSH) para obtenção dos descritores universais. Foram utilizados os descritores controlados em português e inglês, respectivamente: Dispositivos de acesso vascular/ *Vascular access device*; Fístula arteriovenosa/ *Arteriovenous fistula*; Cateteres/ *Catheters*; Cuidados de enfermagem/ *Nursing care*; Diálise renal/ *Renal dialysis*; Terapia de substituição renal/ *Renal replacement therapy*. A seleção dos artigos foi realizada por três pesquisadores de forma independente e cegada, no período de setembro a novembro de 2019, obedecendo rigorosamente aos critérios de inclusão e exclusão definidos no protocolo de pesquisa. O percurso realizado para seleção das publicações incluídas na Revisão Integrativa (RI) está demonstrado na Figura 1, elaborada a partir da recomendação PRISMA (*Preferred Reporting Items for Systematic Reviews and Meta-Analyses*)(13). Ressalta-se que no método da RI não está recomendada a validação do algoritmo de busca dos artigos, contudo o revisor deve deixar claro quais são os critérios de inclusão e exclusão adotados para a elaboração da revisão, como se pode observar neste estudo(10).

Fonte: elaboração própria.

## Análise dos dados

Foram selecionados 10 artigos e utilizou-se um instrumento adaptado pelos autores para auxiliar na leitura crítica dos estudos(14) extraindo características como: país, ano de publicação, delineamento do estudo, número de pacientes, intervenções, desfechos e nível de evidência científica.

Neste último, seguiu-se a classificação recomendada por Melnyk e Fineout-Overholt(15) através da seguinte proposição, nível I: evidências decorrentes de revisão sistemática ou meta-análise de ensaios clínicos randomizados controlados; nível II: evidências de pelo menos um ensaio clínico randomizado controlado bem delineado; nível III: ensaios clínicos bem delineados sem randomização; nível IV: estudos de coorte e de caso-controle bem delineados; nível V: revisão sistemática de estudos descritivos e qualitativos; nível VI: evidências de um único estudo descritivo ou qualitativo; nível VII: opinião de autoridades e/ou relatório de comitês de especialistas.

Elaborou-se um arquivo único de texto contendo o *corpus* textual que foi construído com base nos resultados dos artigos, para que fosse processado e analisado por meio do *software* IRAMUTEQ® (*Interface de R pour* lês *Analyses Multidimensionnelles de Textes et de Questionnaires*), versão 0.7 *alpha* 2, que possibilita diversos processamentos e análises estatísticas de textos. Para a realização da análise dos artigos utilizou-se o método da Classificação Hierárquica Descendente (CHD), também conhecido por modelo de Reinert(16).

Durante o processamento dos dados foi observado que o IRAMUTEQ identificou a divisão do *corpus* em 10 unidades de texto, 102 segmentos de textos, 987 formas distintas e 3.704 ocorrências de palavras no texto. Foram aproveitados 82 segmentos de texto, de um total de 102, ou seja, 80.39% do *corpus* foi utilizado para a análise.

Pela análise CHD, o conteúdo analisado foi categorizado em 7 classes semânticas distintas: Classe 1 com 10 ST (12,2%), Classe 2 com 10 ST (12,2%), Classe 3 com 12 ST (14.63%), Classe 4 com 14 ST (17,07%), Classe 5 com 14 ST (17,07%), Classe 6 com 11 ST (13,41%), Classe 7 com 11 ST (13,41%). As palavras relevantes consideradas foram as com frequência média registrada equivalente a cinco, com valor de p com significância ≥ 0,0001. Representou-se cada classe pelas palavras mais significativas e suas respectivas associações com a classe (qui-quadrado).

Vale ressaltar que as sete classes se encontram divididas em cinco ramificações (A, B, C, D, E) do *corpus* total em análise. O subcorpus A é composto pela Classe 1 (Cuidados com o cateter após a hemodiálise), que se refere aos cuidados praticados pelo paciente para evitar a contaminação do cateter e o reconhecimento e comunicação rápidos de sinais de infecção. O subcorpus B é formado pela Classe 7 (Conhecimento do paciente acerca dos cuidados com a pele e punção da FAV), que contempla o conhecimento dos pacientes sobre os cuidados para punção da FAV e com a pele, como não remover pelos e crostas, e pela Classe 6 (Cuidados realizados pela equipe de enfermagem), que apresenta os cuidados realizados pela equipe de enfermagem com os acessos vasculares no serviço de saúde.

O subcorpus C compõe-se pela Classe 5 (Autocuidado dos pacientes com a FAV), que reúne os cuidados mais praticados pelos pacientes durante o período de maturação da FAV, incluindo a realização de exercícios, evitar o excesso de peso, e a manutenção do controle da pressão arterial. O subcorpus D contém a Classe 4 (Cuidados para evitar a interrupção do funcionamento da FAV), que aborda os cuidados dos pacientes para evitar a interrupção do fluxo da FAV, como não permitir administrar medicamentos, verificar a pressão arterial, e não dormir sobre o braço. A Classe 4 se ramifica no subcorpus E, que contempla a Classe 3 (Cuidados com a FAV após a hemodiálise), e a Classe 2 (Cuidados com a FAV antes da hemodiálise) as quais contemplam os cuidados realizados pelo paciente com a FAV antes e após a hemodiálise, como higienizar o local com água e sabão e evitar movimentos bruscos e força física com os membros superiores.

## Resultados

A interpretação dos resultados foi realizada por meio de avaliação crítica dos estudos revisados, realizando comparações com o conhecimento teórico. Partindo desse pressuposto tornou-se possível identificar as características e inferências dos estudos em análise, sendo utilizada a nomenclatura“A”referente ao artigo, seguido do número arábico para identificação dos estudos.

Os artigos analisados foram publicados entre os anos de 2013 e 2018, com ápice no ano de 2016, o qual concentrou 4 (40%) artigos. Quanto ao país de origem das publicações, 6 (60%) foram realizadas no Brasil, 1 (10%) no Canadá, 1 (10%) nos Estados Unidos (EUA), 1 (10%) no Reino Unido, e 1(10%) no Irã. No que se refere ao nível de evidência das pesquisas, 9 (90%) publicações classificaram-se em nível VI e 1 (10%) no nível II. A caracterização dos artigos analisados encontra-se descrita no Quadro 2.

**Quadro 2:** Caracterização do perfil dos artigos analisados. Brasil, 2019.

Título	País/ Ano	Delineamento/ Número de pacientes	Intervenções	Desfechos	Nível de evidência
(A1) Preservaçã o da fístula arteriovenosa: ações conjuntas entre enfermagem e cliente(17)	Brasil 2013	Descritivo n=17	Não foram realizadas intervenções.	Conhecimento satisfatório dos cuidados para a preservação da fístula arteriovenosa, porém nem todos os cuidados extensivos ao domicílio são seguidos pelos pacientes.	VI
(A2) Fístula arteriovenosa: autocuidado em pacientes com doença renal crônica(18)	Brasil 2013	Descritivo n=60	Não foram realizadas intervenções.	Resultados satisfatórios acerca do autocuidado dos pacientes com a fístula arteriovenosa.	VI
(A3) Survey of home hemodialysis patients and nursing sta_ regarding vascular access use and care(19)	EUA 2015	Descritivo Pacientes com acesso domiciliar para hemodiálise (n = 301) e enfermeiros em treinamento domiciliar (n = 55)	Práticas Geralmente Aceitas para avaliar o treinamento usado pelos enfermeiros para ensinar o cuidado com o acesso vascular e, em seguida, avaliar a adesão dos pacientes ao treinamento.	Os pacientes não realizavam a canulação do acesso de acordo com as práticas geralmente aceitas, nem aderiam ao treinamento. Observou-se relutância dos pacientes em relatar alguns sinais e sintomas de infecção.	VI
(A4) Preoperative assessment and planning of haemodialysis vascular access(20)	Reino Unido 2015	Descritivo n = não se aplica	Três etapas são necessárias para aumentar o número de pacientes elegíveis ao uso de fístula arteriovenosa: processo de atendimento (que inclui a equipe de saúde especializada, educação precoce sobre o acesso vascular e o encaminhamento oportuno da cirurgia); avaliação pré-operatória e estratégia cirúrgica.	Os principais desfechos positivos englobam: educação exitosa sobre os cuidados com o acesso vascular; avaliação pré-operatória criteriosa, sendo obrigatório o exame cuidadoso dos leitos arteriais e venosos, exame físico geral e mapeamento por ultrassom com *doppler* colorido. A estratégia cirúrgica ao levar em consideração a anatomia vascular, fatores clínicos e prognóstico, torna-se então bem-sucedida.	VI
(A5) Pacientes em hemodiálise com fístula arteriovenosa: conhecimento, atitude e prática(21)	Brasil 2015	Descritivo n = 30	Não foram realizadas intervenções.	O conhecimento dos pacientes sobre os cuidados com a fístula arteriovenosa foi inadequado. A redução da ingesta hídrica foi o cuidado mais conhecido entre os pacientes. No entanto, a conduta correta em caso de hematomas na diálise era desconhecida pela maioria dos clientes.	VI
(A6) Hemodialysis Tunneled Catheter Noninfectious Complications(22)	Canadá 2016	Descritivon = não se aplica	Não foram realizadasintervenções.	Descrição das complicações não infecciosas do cateter de hemodiálise.	VI
(A7) Fístula arteriovenosa na perspectiva de pacientes renais crônicos(23)	Brasil 2016	Descritivon=10	Não foram realizadas intervenções.	Os cuidados que os pacientes referiram ter com a fístula arteriovenosa traduziram um conhecimento bastante incipiente e inadequado, independentemente de o início do tratamento ter sido recente ou não.	VI
(A8) Percepção do paciente renal crônico acerca dos cuidados com acessos para hemodiálise (24)	Brasil 2016	Descritivon = 28	Não foram realizadas intervenções.	Os pacientes apresentavam conhecimento prévio acerca dos cuidados com os acessos vasculares. Com relação à atuação da equipe de enfermagem, alguns pacientes não foram orientados adequadamente sobre os cuidados.	VI
(A9) Nursing Strategies for Patients with Chronic Renal Failure Undergoing Maintenance Hemodialysis Treatment by Arteriovenous Fistula(25)	Irã 2016	Ensaio clínicon = 92	Grupo controle (cuidados de enfermagem padrão)Grupo intervenção(estratégias pro_ssionaisde enfermagem interfístula).	As estratégias profissionais de enfermagem direcionadas ao bom funcionamento da fístula podem prolongar o tempo de uso desse acesso, diminuir as complicações e melhorar a qualidade de vida dos pacientes em tratamento hemodialítico.	II
(A10) Pacientes em hemodiálise: importância do autocuidado com a fístula arteriovenosa (26)	Brasil 2018	Descritivo Prospectivon = 32	Não foram realizadas intervenções.	Demonstraram-se lacunas no conhecimento dos pacientes com a fístula arteriovenosa acerca do autocuidado, sendo necessárias mais orientações dadas pelos pro_ssionais de saúde.	VI

Fonte: elaboração própria.

**Figura 2:**
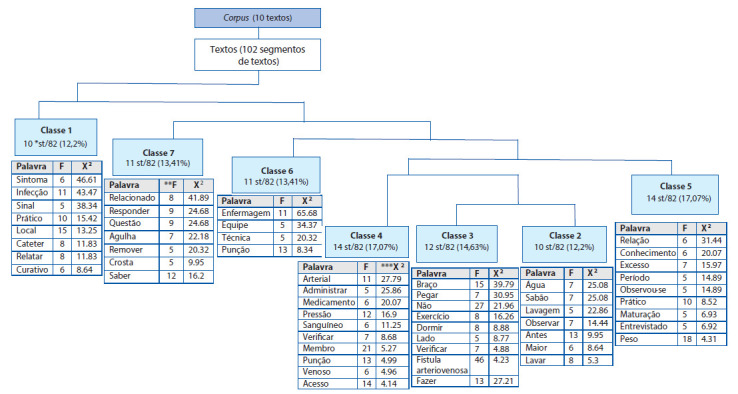
Dendograma da Classificação Hierárquica Descendente. Brasil, 2019.

Por meio das análises no IRAMUTEQ®, o dendograma demonstra as divisões feitas no *corpus* até as classes finais, categorizando as temáticas mais representativas e as palavras mais frequentes em suas respectivas classes (Figura 2).

A seguir, serão descritas as classes obtidas na análise. As sete classes foram nomeadas mediante a identificação e análise de domínios textuais e da interpretação dos significados, sendo: 1 - Cuidados com o cateter após a hemodiálise; 2 - Cuidados com a FAV antes da hemodiálise; 3 - Cuidados com a FAV após a hemodiálise; 4 - Cuidados para evitar a interrupção do funcionamento da FAV; 5 - Autocuidado dos pacientes com a FAV; 6 - Cuidados realizados pela equipe de enfermagem; e 7 - Conhecimento do paciente acerca dos cuidados com a pele e punção da FAV.

Legenda: *st: segmento de texto; **F: frequência da classe; ***X2: valor do qui-quadrado. Fonte: elaboração própria.

Classe 1: Cuidados com o cateter após a hemodiálise

A classe um caracteriza-se por dez segmentos de textos, correspondendo a 12,2% do *corpus*. As palavras dessa classe que tiveram frequência ≥ 5 e foram mais significativas com valor de qui-quadrado (X²) ≥ 3,84 e p < 0,05 foram: sintoma; infecção; sinal; prático; local; cateter; relatar; curativo; e foram extraídas majoritariamente dos artigos três e seis, por ordem de significância. Dessa forma, é possível concluir que pacientes que utilizam o cateter como acesso vascular para a hemodiálise devem estar atentos aos sinais e sintomas de infecções no local da punção, devendo relatar à equipe de saúde o surgimento dessas complicações, bem como desenvolver cuidados específicos no domicílio para preservar o curativo feito no serviço de saúde.

Classe 2: Cuidados com a FAV antes da hemodiálise

A classe dois apresenta dez segmentos de textos que correspondem a 12,2% do *corpus* textual e é uma ramificação da classe quatro e relaciona-se diretamente com a classe três. Os vocábulos dessa classe que tiveram frequência ≥ 5 e foram mais significativas com valor de qui-quadrado (X²) ≥ 3,84 e p < 0,05 foram: água; sabão; lavagem; observar; antes; maior; lavar, retirados dos artigos dez, cinco, um, seis, sete e oito, em ordem de significância.

Classe 3: Cuidados com a FAV após a hemodiálise

Apresentam-se na classe três: 12 segmentos de texto, correspondendo a 14,63% do *corpus* textual e associam-se diretamente à classe dois. Os vocabulários mais frequentes dessa classe foram: braço; pegar; não; exercício; dormir; lado; verificar; fístula arteriovenosa e fazer, que tiveram frequência ≥ 5 e foram mais significativos com valor de qui-quadrado (X²) ≥ 3,84 e p < 0,05, retirados principalmente dos artigos oito, um, sete, dez e dois, de acordo com a ordem de significância.

Classe 4: Cuidados para evitar a interrupção do funcionamento da FAV

Compõem a classe quatro: 14 segmentos de texto, correspondendo a 17,07% do *corpus* textual, associando-se diretamente à ramificação das classes três e dois. Apontam-se os vocábulos predominantes: arterial; administrar; medicamento; pressão; sanguíneo; verificar; membro; punção; venoso; acesso. Obtiveram frequência ≥ 5 e foram mais significativos com valor de qui- quadrado (X²) ≥ 3,84 e p < 0,05, retirados principalmente dos artigos sete, dez, um, cinco e dois, por ordem de significância.

Classe 5: Autocuidado dos pacientes com a FAV

Essa classe é composta por 14 segmentos de texto, o que corresponde a 17,07% do *corpus* textual e está associada às classes dois, três e quatro. Os vocábulos que mais apareceram foram: relação; conhecimento; excesso; período; observou-se; prático; maturação; entrevistado e peso, em que obtiveram uma frequência 5 e foram mais significativos apresentando qui-quadrado (X²) ≥ 3,84 e p < 0,05, retirados principalmente dos artigos cinco, dez, sete, dois e oito, em ordem de significância.

Classe 6: Cuidados realizados pela equipe de enfermagem

Incluem-se, na classe seis, 11 segmentos de texto, correspondendo a 13,41% do *corpus* textual. Elencam-se os vocabulários com frequência ≥ 5, mais significativos com valor de qui-quadrado (X²) ≥ 3,84 e p < 0,05 desses segmentos: enfermagem; equipe; técnica; punção, retirados principalmente dos artigos oito, nove, um, dez e quatro, de acordo com a significância.

Classe 7: Conhecimento do paciente acerca dos cuidados com a pele e punção da FAV Apresentam-se, na classe sete, 11 segmentos de texto, correspondendo a 13,41% do *corpus* textual. Os vocabulários que tiveram frequência ≥ 5 e foram mais significativas com valor de qui-quadrado (X²) ≥ 3,84 e p < 0,05 da respectiva classe foram: relacionado; responder; questão; agulha; remover; crosta; saber, extraídos predominantemente dos artigos dez, três, quatro e nove.

## Discussão

Evidencia-se, pelos conteúdos apreendidos das classes, que os cuidados com os acessos vasculares para hemodiálise estão organizados em cuidados realizados no serviço de saúde e no domicílio, de forma complementar. Essa contextualização é importante, tendo em vista que os cuidados para manter o acesso venoso pérvio devem ser tomados tanto pelos profissionais da saúde quanto pelo paciente. A definição dos sujeitos do cuidado e de onde ocorrem suas respectivas atuações contribui para o estabelecimento de suas respectivas responsabilidades(27).

Os pacientes corretamente orientados quanto às recomendações do tratamento tornam-se corresponsáveis pela prevenção das complicações ou perdas do acesso vascular para hemodiálise, contribuindo com a eficiência terapêutica. Um estudo transversal reafirma também que pacientes devidamente instruídos e munidos de conhecimento adequado realizam medidas preventivas com mais frequência, resultando em menor taxa de complicações e aumento da taxa de funcionamento do seu acesso(28).

Uma parte dos estudos analisados nesta revisão apresenta os cuidados principais realizados antes e após a sessão de HD, quer seja no serviço de saúde, quer seja no domicílio. Dentre os cuidados com o cateter venoso central apresentados nos estudos, destacam-se aqueles realizados pelo paciente ao deixar a clínica, sendo então o responsável principal após a sessão de HD pela manutenção do curativo limpo e seco, evitando infecções.

O cateter requer cuidados específicos, principalmente relacionados à manipulação, assim, para a prevenção das complicações infecciosas é preferível que seja substituído pela fístula o mais rápido possível. É recomendável que o paciente evite dormir sobre o cateter, não o manipule nem o molhe e, para isso, deve proteger o curativo durante o banho. Outrossim, além de manter o curativo limpo e seco, o paciente deve observar sinais de umidade, dor, sangramento, febre e/ ou secreção no local, devendo retornar ao serviço e comunicar à equipe que presta a assistência(29).

Destaca-se a importância do autocuidado do paciente com o cateter no domicílio, sendo necessária a sua devida orientação para que seja capaz de identificar os sinais de infecção e procure o serviço de saúde em tempo oportuno. O reconhecimento precoce dos sinais flogísticos, além de conferir autonomia ao sujeito com os cuidados relativos ao seu acesso vascular, contribui para a prevenção de infecções, como sepse e endocardite, já que o acesso vascular central permite o acesso rápido dos micro-organismos à circulação sistêmica(30).

Evidencia-se na revisão que os cuidados com a fístula arteriovenosa foram responsáveis pela construção de cinco das sete classes representadas nos resultados, demonstrando que esse acesso vascular tem grande representatividade no tratamento do paciente com DRC. Dentre as evidências nas classes acerca da FAV foram destacados os cuidados realizados antes e após a HD, cuidados realizados para evitar a interrupção do seu funcionamento, e até mesmo ressaltou-se o conhecimento do paciente acerca dos cuidados com a punção da FAV e com a pele sobre a FAV após a hemodiálise.

Os cuidados com a FAV iniciam-se ainda na fase de preparo da pele, antes da punção venosa pelo profissional de enfermagem, contemplando a higienização do braço com água e sabão neutro ou outra solução antisséptica. Esses cuidados são muito importantes e justificam-se pelas constantes interrupções cutâneas provocadas pelas punções venosas e pelo ambiente propício à ocorrência de infecções. A elevada frequência desse cuidado nos artigos analisados demonstra a sua ampla difusão nos serviços de saúde, sendo corroborado por um estudo transversal que demonstrou o conhecimento de 100% dos pacientes em terapia hemodialítica, acerca da necessidade de executar essa ação(26).

Durante a sessão de HD o paciente em uso da FAV também deve ser instruído pelo enfermeiro a observar as situações em que o seu acesso vascular não deve ser utilizado, tais como a administração de medicamentos endovenosos, a aferição de pressão arterial e a coleta de sangue. Dessa forma, podem ser evitadas complicações como hematoma, estenose, trombose, isquemia e infecção(31). Ademais, o conhecimento do paciente acerca dos cuidados com a punção da FAV é importante, pois a observação do rodízio de punção auxilia a evitar complicações aneurismáticas(32).

Após a sessão de HD, os cuidados com o acesso vascular devem ser continuados no domicílio, pois nesse ambiente a pessoa em tratamento hemodialítico assume ainda mais seu protagonismo, devendo demonstrar comprometimento com o tratamento através do autocuidado. Desse modo, precisa realizar ações para o correto desenvolvimento e a manutenção da FAV, a partir dos exercícios diários com a bola de borracha, percepção do frêmito e observação da presença dos sinais cardinais da inflamação, exemplificados por eritema, dor, calor e edema(33),(34).

Intervenções comportamentais também são recomendadas, tais como não comprimir, traumatizar ou deitar-se sobre o membro com a fístula, evitar esforços ou segurar grandes cargas, bem como não remover a crosta formada no local da punção. Hemorragias também devem ser verificadas e, em caso de ocorrência, o paciente deve comprimir o local da FAV e solicitar o atendimento de emergência. Em caso de hematoma local, deve ser orientada a aplicação de compressas frias nas primeiras 24 horas e, em seguida, a aplicação de compressas quentes. A ingesta hídrica também deve ser controlada, haja vista que excessos podem ocasionar hipotensão intradialítica e falha do acesso vascular(35).

Observa-se que no ambiente domiciliar o paciente está sujeito ao enfrentamento de limitações decorrentes da terapia implementada, por isso, deve ser auxiliado a ultrapassar tais dificuldades por meio de readaptações em sua rotina, com suporte da equipe de saúde e de seus familiares enquanto redes de apoio. Compreende-se que, proporcionalmente, quanto maior o nível de gerência da própria saúde e sua capacidade de autocuidado, menor é a influência negativa da patologia no cotidiano de pacientes com DRC, em que o mesmo deve ter ciência das consequências relacionadas à negligência dos cuidados, como possíveis infecções e falhas no tratamento(36).

No que tange aos cuidados realizados no serviço de saúde com os acessos vasculares, a análise do conteúdo dos artigos resultou em uma classe que ressalta os cuidados realizados pela equipe de enfermagem. Esse resultado reafirma a importância do enfermeiro na assistência direta ao paciente com DRC e sua contribuição para que o paciente realize os cuidados adequados com o acesso vascular.

O enfermeiro deve capacitar os pacientes e orientá-los a reconhecer os cuidados necessários com o acesso vascular, a fim de facilitar a adesão às novas rotinas impostas pelo tratamento por meio do ensino do autocuidado(33). Acredita-se que a enfermagem assume importante papel no processo de ensino-aprendizagem do paciente com DRC, ao considerá-lo como protagonista do seu próprio tratamento e utilizar materiais de ensino ilustrados e com informações de fácil compreensão.

Alguns materiais instrucionais produzidos por enfermeiros têm sido pioneiros nessa temática, como as cartilhas educativas(30),(37). Esses materiais podem ser consultados pelos pacientes em caso de dúvidas, assim como pelos familiares que podem vivenciar momentos de insegurança para realizar os cuidados no contexto domiciliar.

Depreende-se, assim, que as intervenções educativas realizadas pelo enfermeiro nefrologista no cuidado ao paciente renal crônico podem favorecer a compreensão da doença e a adesão ao tratamento, melhorando a qualidade dos cuidados realizados com os acessos e, por conseguinte, reduzir a morbimortalidade dos pacientes(38).

## Conclusão

Conclui-se que os cuidados com os acessos vasculares mais frequentes se relacionam com a fístula arteriovenosa, demonstrando sua grande representatividade no tratamento do paciente com doença renal crônica. Avalia-se que os cuidados encontrados colocam o paciente como protagonista e requerem o acompanhamento direto do enfermeiro nefrologista.

Os cuidados realizados pela equipe de enfermagem também se destacam na revisão no tocante às orientações acerca da doença, limitações do tratamento, sinais flogísticos e manutenção dos acessos funcionantes e pérvios.

Considera-se como limitação do estudo a seleção dos artigos disponíveis eletrônica e gratuitamente, publicados em determinadas bases de dados e apenas nos idiomas português, inglês ou espanhol, critérios que podem ocultar estudos que contemplem outros critérios de inclusão relacionados aos descritores utilizados. Ademais, pontua-se outra limitação como o baixo nível de evidência dos estudos, o que pode ser oriundo do próprio método da RI, pois permite a inclusão de múltiplos estudos com diferentes delineamentos de pesquisas.

O estudo apresenta uma síntese de cuidados relacionados ao acesso vascular para hemodiálise que devem ser realizados tanto nos serviços de saúde quanto no domicílio, podendo servir de embasamento teórico-científico para a elaboração de Procedimento Operacional Padrão (POP) em serviços que ofertam esse tratamento, para o aperfeiçoamento dos profissionais envolvidos na assistência ao paciente com doença renal crônica, e nos momentos de realização de educação permanente e continuada das equipes. Além disso, também pode fornecer suporte para a elaboração de tecnologias educativas que podem ser utilizadas na assistência à saúde.

Ressalta-se que a síntese do conhecimento proporcionada nesta revisão de literatura foi utilizada para a elaboração de uma cartilha educativa já validada por especialistas e por pacientes que realizam hemodiálise quanto ao conteúdo e à aparência. Os resultados do processo de validação da cartilha serão apresentados em estudo posterior, mediante a organização da versão final do material com as sugestões dos especialistas e do público-alvo.
